# Nocturnal Sleep Problems Mediate the Impact on Quality of Life of Early Morning Off in Parkinson's Disease

**DOI:** 10.3389/fnagi.2021.681773

**Published:** 2021-08-06

**Authors:** Yu Zhang, Zi en Zhang, De Shi, Yi Zhao, Lihong Huang, Yanxin Zhao, Hui Wang, Jing Zhao, Feng Wang, Chaorong Zhao, Shan Gao, Wenshi Wei, Dongya Huang, Zhen guo Liu

**Affiliations:** ^1^Department of Neurology, Xinhua Hospital Affiliated to Shanghai Jiao Tong University School of Medicine, Shanghai, China; ^2^Department of Neurology, Xuhui Center Hospital in Shanghai, Shanghai, China; ^3^Department of Neurology, Jingan District Zhabei Central Hospital, Shanghai, China; ^4^Department of Neurology, Tenth People's Hospital Affiliated to Tongji University, Shanghai, China; ^5^Department of Neurology, Dahua Hospital of Xu hui District, Shanghai, China; ^6^Department of Neurology, Minhang Hospital, Fudan University, Shanghai, China; ^7^Department of Neurology, Seventh People's Hospital of Shanghai University of Traditional Chinese Medicine, Shanghai, China; ^8^Department of TCM, Shanghai Putuo District Hospital of Traditional Chinese Medicine, Shanghai, China; ^9^Department of Neurology, Shanghai Jiao Tong University Affiliated the Sixth People Hospital, Shanghai, China; ^10^Department of Neurology, Huadong Hospital, Fudan University, Shanghai, China; ^11^Department of Neurology, East Hospital, Tongji University School of Medicine, Shanghai, China

**Keywords:** sleep disturbance, early morning off, quality of life, Parkinson's disease, China

## Abstract

**Background:** Early morning off (EMO) refers to off-states in the morning in people diagnosed with Parkinson's disease (PwPD). This study determined the clinical manifestations of EMO and the association with nocturnal sleep problems and quality of life (QOL) in Chinese PwPD.

**Methods:** In this multicenter, observational, cross-sectional study, data concerning the clinical manifestations of EMO were collected from PwPD in Shanghai by questionnaire. The stepwise logistic regression was performed to analyze the potential risk factors, as well as whether EMO was an independent risk factor for functional dependency in daily life. The mediation analyses were conducted to evaluate whether nocturnal sleep problems might mediate the association between EMO and the QOL.

**Results:** Among the 454 subjects evaluated, EMO occurred in 39.43% of PwPD across all disease stages. The prevalence of EMO increased as the Hoehn and Yahr stage increased and was observed in 35.60% of patients in stages 1–2.5 and 48.85% of patients in stages 3–5. EMO was associated with non-motor symptoms (NMSs). The predominant NMSs associated with EMO were nocturnal sleep problems (98.90%), mood/cognition impairment (93.90%), decreased attention/memory (91.60%), gastrointestinal symptoms (91.60%), and urinary urgency (90.50%). The QOL of PwPD with EMO was significantly reduced (*P* < 0.001). Moreover, nocturnal sleep problems might partially mediate this relationship (indirect effect: β = 13.458, 95% boot CI: 6.436, 22.042).

**Conclusion:** PwPD have EMO throughout all stages of the disease. Patients with EMO have severe motor symptoms and NMSs. EMO decreases the QOL in PwPD and this relationship is partially mediated by nocturnal sleep problems. In light of these findings, it is suggested that recognition and appropriate treatment of EMO and nocturnal sleep problems could improve the management of PwPD.

## Introduction

Parkinson's disease (PD) is a common movement disorder. Clinical manifestations of PD are heterogeneous and cover a broad spectrum of manifestations ranging from motor symptoms to non-motor symptoms (NMSs). These include bradykinesia, rigidity, tremor, cognitive impairments, and mood disorders (Ascherio and Schwarzschild, 2016; Tysnes and Storstein, 2017). Several NMSs of PD predate the movement symptoms by decades. Early morning off (EMO) is a motor complication in people diagnosed with Parkinson's disease (PwPD) (Chapuis et al., 2005). Since there was no established definition of EMO, the presence of EMO was identified in patients who present with poor motor or NMSs on morning waking, while related symptoms could be alleviated after taking the first dosage of the dopaminergic drug. EMO is related to the concentration of anti-Parkinson's medications in the serum, which is at a low level during the night and can be relieved until the first dose of dopaminergic therapy; sometimes it takes longer than usual to restore the ON-state from the first dose of dopaminergic therapy (Stocchi et al., 2019). Rotigotine was an efficacious treatment to reduce early morning motor functioning, reduced sleep disturbances, and improved quality of life (QOL), as it has relevant clinical implications and recommendations (Ray Chaudhuri et al., 2016).

Patients with EMO complain of slowness or stiffness in the early morning and experience an “off” state during the remaining daytime hours. It has been confirmed that EMO has a substantial impact on QOL by A Rizos group (Rizos et al., 2014). Occasionally, patients report that EMO-induced dystonia with pain causes nocturnal sleep problems and imposes additional burdens on caregivers (Rizos et al., 2016; Han et al., 2020). Nocturnal sleep problems are common NMSs in PwPD that negatively impact the QOL of patients (Stefani and Högl, 2020). EMO has been reported to be associated with nocturnal sleep problems and reflects the nocturnal decline in dopamine levels with insufficient night-time storage (Stocchi et al., 2019). However, objective evidence of how nocturnal sleep problems correlated or regulates the impact of EMO on QOL is limited. Exploring the effects of nocturnal sleep problems on this relationship may reveal important implications for early interventions designed to enrich and prolong the QOL of PwPD.

In this multicenter, observational, cross-sectional study, we determined the clinical characteristics of EMO in PwPD and verified the association with nocturnal sleep problems and QOL, to provide a comprehensive understanding of the EMO phenomenon in Chinese PwPD.

## Methods

### Study Design and Participants

We conducted a multicenter, observational, outpatient-based, cross-sectional study that was approved by the Research Ethics Committee of Xin Hua Hospital affiliated to Shanghai Jiao Tong University School of Medicine and the Ethics Committees of other sites. After obtaining informed consent, PwPD were consecutively recruited and diagnosed according to the Movement Disorder Society PD criteria (Postuma et al., 2015). Patients with secondary parkinsonism, stroke, brain tumor, or an alternative cause for parkinsonism symptoms were excluded. We did not set an exclusion criterion based on sleep.

### Clinical Assessment of the Motor Symptoms and NMSs of PwPD

Patients were recruited from 12 sites. This study screened 462 patients and enrolled 454 patients. Eight patients were excluded as they did not meet the inclusion criteria. A structured interview for clinical and demographic variables was performed. All PwPD were evaluated during the medication “on” period. The symptoms of PwPD were measured with the following scales: the Unified Parkinson's Disease Rating Scale (UPDRS), modified Hoehn–Yahr (HY) stage, Mini-Mental State Examination (MMSE), and the Non-Motor Symptom Scale (NMSS) (Chaudhuri et al., 2007). The nocturnal sleep problems of patients were assessed with the Parkinson's Disease Sleep Scale (PDSS). The QOL of patients was assessed with the Parkinson's Disease Questionnaire-39 (PDQ-39).

Since there is no established definition of EMO in PwPD, the presence of EMO was identified using a two-step process during a structured clinical interview in the first instance, as described by Rizos et al. (Rizos et al., 2014).

The interview included specific questions based on the definition of the wearing-off state upon morning awakening, while related symptoms could be alleviated after taking the first dosage of the dopaminergic drug and supplemented by the following: (1) how the patient is upon awakening (morning “on” vs. morning “off” stages), (2) response to UPDRS item 35 [presence of early morning dystonia (historical information)], (3) response to PDSS item 14 (do you feel tired and sleepy after awakening in the morning?), and (4) whether these symptoms improve after a morning dose of dopaminergic drug (Rizos et al., 2014).

### Statistical Analysis

The statistical analysis was performed using SPSS 26.0 (IBM. Corp., Armonk, New York, USA). Categorical variables were compared using the Pearson's chi-square test, the Mann–Whitney *U* test, or Student's *t*-test. A binary logistic regression model was performed to evaluate the factors that could independently associate with EMO. A linear regression of PDQ-39 total scores was performed with different variables. The level of significance was set at *P* < 0.05. Odds ratios (ORs) are presented with 95% CIs.

Mediation analyses were conducted with the SPSS mediation modeling software, PROCESS, to determine whether the severity of nocturnal sleep problems mediated the association between EMO and QOL. The analysis considered the total effect of EMO (with or without EMO) on QOL (PDQ-39) and the indirect effect mediated by PDSS total scores. A bootstrap estimation approach with 5,000 samples was used to measure the indirect effect with 95% CIs. The indirect effect was considered significant when the 95% CIs did not contain zero.

## Results

A total of 454 PwPD were enrolled in this study. The mean age of the patients was 68.97 ± 8.620 years and 56.17% were males (Table 1). The mean disease duration of PwPD was 5.954 ± 4.762 years. The mean modified HY stage was 2.269 ± 0.874 (stage 1–2.5, *n* = 323; stage 3–5, *n* = 131). The mean UPDRS total score and UPDRS part III scores were 51.02 ± 29.340 and 28.10 ± 16.506, respectively. The mean NMS total score was 58.36 ± 48.676, and every component NMS score (from I to IX) was presented in Table 1.

**Table 1:** Clinical characteristics of all PD patients with and without EMO groups.

**Mean** **±** **SD**				
**Variable**	**With EMO (** ***n*** **=** **179)**	**Without EMO (** ***n*** **=** **275)**	**Total (** ***n*** **=** **454)**	***P*** **-value**
Male (%)	54.2	57.45	56.17	0.493
Age at study, y	70 ± 9.787	68.23 ± 7.693	68.97 ± 8.620	0.008
Age of PD onset, y	63.81 ± 10.945	62.04 ± 9.711	62.73 ± 10.239	0.063
Disease duration, y	6.164 ± 5.449	5.818 ± 4.266	5.954 ± 4.762	0.979
Modified Hoehn-Yahr stage	2.534 ± 0.978	2.096 ± 0.751	2.269 ± 0.874	< 0.001
UPDRS Total	70.44 ± 32.154	38.37 ± 18.57	51.02 ± 29.340	< 0.001
UPDRS III	36.68 ± 18.909	22.52 ± 11.78	28.10 ± 16.506	< 0.001
PDSS total score	23.84 ± 9.903	11.40 ± 7.132	16.3 ± 10.313	< 0.001
PDSS-1	2.15 ± 1.134	1.55 ± 1.205	1.79 ± 1.212	< 0.001
PDSS-2	1.90 ± 1.232	0.92 ± 1.257	1.30 ± 1.336	< 0.001
PDSS-3	2.26 ± 1.250	1.84 ± 1.413	2.0 ± 1.365	0.002
PDSS-4	1.59 ± 1.003	0.48 ± 0.905	0.92 ± 1.090	< 0.001
PDSS-5	1.45 ± 0.989	0.29 ± 0.652	0.74 ± 0.981	< 0.001
PDSS-6	1.72 ± 1.190	1.06 ± 1.182	1.32 ± 1.228	< 0.001
PDSS-7	0.98 ± 1.104	0.28 ± 0.693	0.56 ± 0.901	< 0.001
PDSS-8	2.46 ± 1.143	2.11 ± 1.412	2.25 ± 1.323	0.005
PDSS-9	1.82 ± 1.229	0.61 ± 1.080	1.09 ± 1.283	< 0.001
PDSS-10	1.28 ± 1.045	0.31 ± 0.757	0.70 ± 1.001	< 0.001
PDSS-11	1.39 ± 0.996	0.56 ± 0.900	0.89 ± 1.021	< 0.001
PDSS-12	1.22 ± 1.052	0.22 ± 0.607	0.61 ± 0.949	< 0.001
PDSS-13	1.52 ± 1.177	0.49 ± 0.998	0.90 ± 1.183	< 0.001
PDSS-14	2.18 ± 1.066	0.67 ± 1.093	1.26 ± 1.310	< 0.001
PDSS-15	1.16 ± 1.032	0.22 ± 0.636	0.59 ± 0.935	< 0.001
NMS Total	88.09 ± 58.448	39.00 ± 27.283	58.36 ± 48.676	< 0.001
NMS-I	3.44 ± 3.892	1.43 ± 2.623	2.22 ± 3.329	< 0.001
NMS-II	14.21 ± 10.132	8.74 ± 7.467	10.89 ± 9.012	< 0.001
NMS-III	20.58 ± 18.101	8.03 ± 10.588	12.97 ± 15.293	< 0.001
NMS-IV	3.29 ± 4.916	0.90 ± 2.240	1.84 ± 3.727	< 0.001
NMS-V	9.32 ± 8.227	3.92 ± 4.864	6.05 ± 6.919	< 0.001
NMS-VI	10.12 ± 8.175	4.71 ± 5.209	6.84 ± 7.048	< 0.001
NMS-VII	10.20 ± 8.801	5.86 ± 7.377	7.58 ± 8.238	< 0.001
NMS-VIII	7.35 ± 9.102	0.70 ± 3.390	3.32 ± 7.078	< 0.001
NMS-IX	9.30 ± 8.631	4.84 ± 5.457	6.60 ± 7.213	< 0.001
MMSE score	21.80 ± 6.271	26.85 ± 3.422	24.86 ± 5.351	< 0.001
PDQ-39 score	63.98 ± 31.520	25.68 ± 20.207	40.78 ± 31.438	< 0.001
Total LED, mg	631.078 ± 383.730	519.636 ± 330.735	563.575 ± 356.363	0.003
L-dopa medication, mg	500.150 ± 250.496	426.872 ± 207.557	457.931 ± 229.331	0.003
DA medication, mg	21.159 ± 38.517	32.159 ± 44.483	27.822 ± 42.530	0.252
MAO-B inhibitor, mg	10.00 ± 4.364	7.554 ± 3.378	8.210 ± 3.800	0.018

*PD, Parkinson's disease; EMO, early morning off; PDSS, Parkinson's disease sleep scale; PDQ, Parkinson's disease questionnaire; UPDRS, unified Parkinson's disease rating scale; NMS, non-motor symptom; MMSE, minimum mental state examination; DA, dopamine agonists; MAO-B, monoamine oxidase-B; LED, levodopa equivalent dose*.

**Figure 1:**
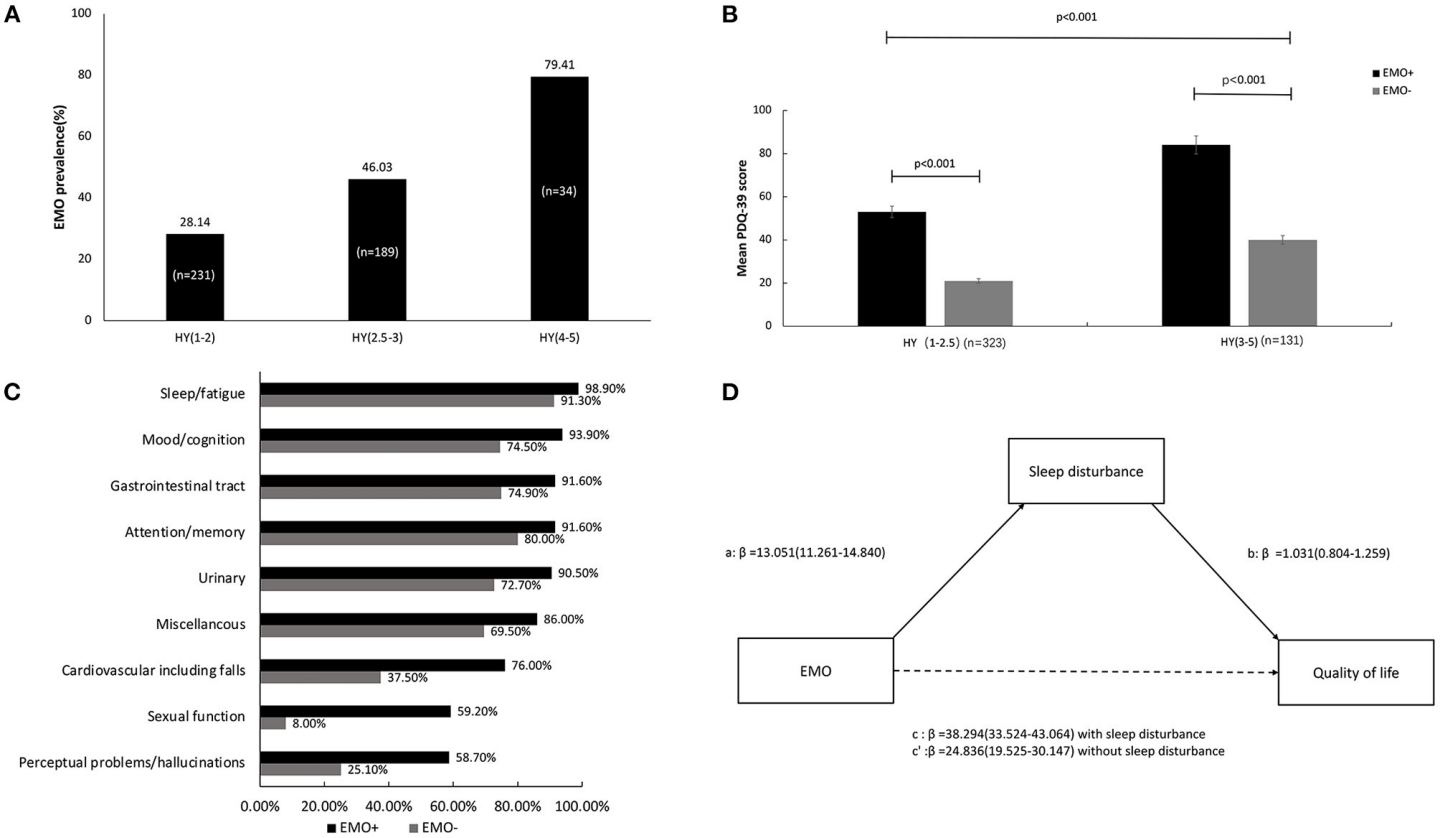
The clinical manifestations of EMO and the association with nocturnal sleep problems and quality of life (QOL) in Chinese PWPD. **(A)** The prevalence of EMO in different stages of HY. **(B)** The comparison of mean PDQ-39 scores in different stages of HY between the EMO and non-EMO groups. **(C)** The proportion of non-motor symptoms between the EMO and non-EMO groups. **(D)** The mediation effect of nocturnal sleep problems on the association between EMO and QOL.

The prevalence of patients with EMO was 39.43% (*n* = 179). The EMO group had a tendency to be older at the time of this study (70 ± 9.787 years vs. 68.23 ± 7.693 years, *P* = 0.008). Disease duration and family history did not exhibit significant differences between the two groups (Table 1). EMO was present in 28.14% of patients in stage 1–2, 46.03% of patients in stage 2.5–3, and 79.41% of patients in stage 4–5 (Figure 1A). PwPD with EMO were more likely to have a lower QOL, especially in advanced HY stages (Figure 1B). The reason for the distinction between stage 2.5 and below and stage 3 is based on the Chinese guidelines for the treatment of PD (fourth edition) [Chinese guidelines for the Treatment of Parkinson′s disease (fourth edition)., 2020]. The EMO group also had a higher dose of levodopa-equivalent dose (LED) (631.078 ± 383.730 vs. 519.636 ± 330.735, *P* = 0.003), higher dose of L-dopa medication (500.150 ± 250.496 vs. 426.872 ± 207.557, *P* = 0.003), higher dose of monoamine oxidase-B (MAO-B) inhibitor (10.00 ± 4.364 vs. 7.554 ± 3.378, *P* = 0.018), higher UPDRS-III scores (36.68 ± 18.909 vs. 22.52 ± 11.78, *P* < 0.001), higher NMS scores (88.09 ± 58.448 vs. 39.00 ± 27.283, *P* < 0.001), higher PDSS scores (23.84 ± 9.903 vs. 11.40 ± 7.132, *P* < 0.001), lower MMSE scores (21.80 ± 6.271 vs. 26.85 ± 3.422, *P* < 0.001), and higher PDQ-39 scores (63.98 ± 31.520 vs. 25.68 ± 20.207, *P* < 0.001). Also, there were statistical differences in each item in PDSS and NMS between two groups (Table 1). The percentage of NMSs between the two groups was significantly different. The prevalence of NMSs in the EMO group was higher in contrast to the non-EMO group, such as sleep disturbance/fatigue (98.90 vs. 91.30%, *P* < 0.001), depressed mood/cognitive impairment (93.90 vs. 74.50%, *P* < 0.001), decreased attention/memory (91.60 vs. 74.90%, *P* < 0.001), gastrointestinal symptoms (91.60 vs. 80.00%, *P* < 0.001), urinary urgency (90.50 vs. 72.70%, *P* < 0.001), cardiovascular symptoms (76.00 vs. 37.5%, *P* < 0.001), sexual dysfunction (59.20 vs. 8.00%, *P* < 0.001), perceptual problems/hallucinations (58.70 vs. 25.10%, *P* < 0.001), and miscellaneous symptoms (86.00 vs. 69.50%, *P* < 0.001) (Figure 1C).

The logistic regression analysis of EMO using a likelihood ratio forward selection showed that several factors contributed to EMO, including PDSS scores (OR = 1.196, CI = 1.156–1.238, *P* < 0.001), PDQ-39 scores (OR = 1.045, CI = 1.031–1.060, *P* < 0.001), UPDRS III scores (OR = 1.048, CI = 1.030–1.066, *P* < 0.001), NMS total scores (OR = 1.016, CI = 1.008–1.024, *P* < 0.001), MMSE scores (OR = 0.887, CI = 0.837–0.940, *P* < 0.001), and total LED (OR = 0.999, CI = 0.998–1.000, *P* = 0.001; Table 2). We further performed the regression analysis on the sub-items of NMSS and PDSS with EMO to determine which items in NMSS and PDSS are the core driving factors of EMO. The results showed that PDSS-5 (OR = 3.282, CI = 2.267–4.752, *P* < 0.001), PDSS-13 (OR = 1.627, CI = 1.269–2.087, *P* < 0.001), PDSS-14 (OR = 2.571, CI = 1.968–3.359, *P* < 0.001), PDSS-15 (OR = 2.108, CI = 1.443–3.081, *P* < 0.001), NMS-II (OR = 0.923, CI = 0.884–0.963, *P* < 0.001), and NMS-VIII (OR = 1.142, CI = 1.083–1.204, *P* < 0.001) were core driving factors of EMO (Table 3).

**Table 2:** Multivariate binary logistic regression analyses of the factors associated with EMO.

**Variables**	**OR (95% CI)**	***P*-value**
PDSS score	1.196 (1.156–1.238)	<0.001
PDQ-39 score	1.045 (1.031–1.060)	<0.001
MMSE	0.887 (0.837–0.940)	<0.001
UPDRS III	1.048 (1.030–1.066)	<0.001
NMS Total	1.016 (1.008–1.024)	<0.001
Total LED	0.999 (0.998–1.000)	0.001

*PDSS, Parkinson's disease sleep scale; PDQ, Parkinson's disease questionnaire; MMSE, minimum mental state examination; UPDRS, unified Parkinson's disease rating scale; NMS, non-motor symptom; LED, levodopa equivalent dose*.

**Table 3:** Multivariate binary logistic regression analyses of sub-items in NMS and PDSS associated with EMO.

**Variables**	**OR (95% CI)**	***P*-value**	**Standardized Coefficients**
PDSS-5	3.282 (2.267–4.752)	<0.001	0.643
PDSS-13	1.627 (1.269–2.087)	<0.001	0.318
PDSS-14	2.571 (1.968–3.359)	<0.001	0.682
PDSS-15	2.108 (1.443–3.081)	<0.001	0.385
NMS-II	0.923 (0.884–0.963)	<0.001	0.403
NMS-VIII	1.142 (1.083–1.204)	<0.001	0.519

*EMO, early morning off; PDSS, Parkinson's disease sleep scale; NMS, non-motor symptom*.

When the PDSS scores were entered as mediators of the EMO and PDQ-39, the total effect was significant (β = 38.294, *P* < 0.001), the indirect effect was significant (β = 13.458, *P* < 0.001), and the direct effect remained significant (β = 24.836, *P* < 0.001). Therefore, we inferred that nocturnal sleep problems partially mediated the effect of EMO on QOL (Figure 1D).

## Discussion

Previous studies have focused on the effects of daytime wearing-off on PwPD (Stacy et al., 2005; Stacy, 2010). In this study, we found that EMO was observed in all stages of PD, even in 28.14% of patients in stages 1–2. In light of these findings, we suggested that EMO should be the focus of attention during the management of all stages of PD. The factors that are associated with EMO are complicated. According to the statistical results in our cohort, we showed that motor symptoms are associated with EMO. There were deteriorating motor symptoms and a more advanced HY stage in the EMO group than the non-EMO group, which is consistent with the results suggested by Onozawa et al. (Rizos et al., 2016). Therefore, patients with EMO should undertake medical intervention to counteract the disease symptoms. We also found that the total LED was a factor associated with EMO. It might be caused by people with worse EMO have worse motor symptoms and so are on higher LED dosage. Besides, the patients with EMO take a higher dosage of l-dopa medication than the non-EMO group, since they need to take a high dose of l-dopa medication to deviate their symptoms and control their condition. This study also showed that the dose of MAO-B inhibitor was higher in the EMO group than the non-EMO group; however, this study did not further explore which type of MAO-B inhibitor was more effective. The other types of MAO-B inhibitor, such as Rasagiline, were rarely used in our research than Selegiline, which was widely taken in PwPD in China. But some studies proved that Rasagiline was efficacious in reducing “off” time and in improving EMO but also some NMS, thus enhancing the therapeutic approach to PD (Stocchi et al., 2015). The most used dopamine agonists (DA) recorded in this study was Pramipexole, whereas new types of DA, such as Ropinirole and Rotigotine, were barely used in clinical practice in China. In this study, there was no difference in the dose of DA medication between the two groups. Therefore, the impact of varieties of anti-PD medication on EMO needs a long-term clinical follow-up to clarify.

Another factor associated with EMO was NMSs. The EMO group had higher NMS scores than the non-EMO group (Table 1). Common NMSs include sleep disturbance/fatigue (98.90%), depression (93.90%), decreased attention/memory (91.60%), gastrointestinal symptoms (91.60%), and urinary urgency (90.50%). Rizos et al. (Rizos et al., 2014) reported that the common NMSs consist of urination (61.3%), anxiety (49.7%), drooling (46.6%), pain (46.6%), low mood (45.5%), and paresthesia (42.6%). Hence, the NMSs of the EMO group were wide-ranging and should be evaluated. Moreover, nocturnal sleep problems, such as an NMS, were more significant in the EMO group. Although the EMO group had higher NMS scores of each item than the non-EMO group, our results cannot directly conclude that which NMS can distinguish patients with EMO and non-EMO, which requires prospective studies to prove. Moreover, further analysis showed that sexual dysfunction (NMS-VIII) and cognitive impairment (NMS-II) in the NMS are the main factors that affect EMO. Therefore, our results indicated that EMO could be a clinical marker of, not only sleep problems but also possibly a “dysautonomia/cognitive” aggressive phenotype and help raise awareness for clinical practice implications.

In this study, we verified that EMO significantly decreases the QOL of PwPD and that this is partially mediated by nocturnal sleep problems. Our results showed that the EMO group had higher PDQ-39 scores, thus they have an inferior QOL compared with the non-EMO group. Nocturnal sleep problems can also have a negative impact on QOL (Gómez-Esteban et al., 2011; Schrempf et al., 2014), and this study revealed that nocturnal sleep problems were the factors associated with EMO. We, therefore, analyzed the effect of nocturnal sleep problems and EMO on QOL. Nocturnal sleep problems on QOL were revealed to have a mediating effect according to this study. The QOL of patients with EMO could be improved not only by the management of EMO but also by the treatment of nocturnal sleep problems. At the same time, we should monitor the sleep quality of PD patients with EMO who do not suffer from nocturnal sleep problems and be alert to their occurrence. Therefore, both motor and NMSs of patients with EMO should be evaluated.

There were several limitations in this study. First, this study was retrospective, so the causality between EMO, nocturnal sleep problems, and QOL could not be confirmed. It is crucial to conduct prospective studies for verification. Second, the patients were evaluated at their “on” state, which largely required them to recall symptoms, which might lead to recall bias. Finally, due to the lack of a standard for accurate quantitative evaluation of EMO, there is a margin of error in the accuracy assessment.

This cross-sectional non-interventional study provides prevalence information regarding EMO phenomena in Chinese PwPD. This study provided a good characterization of motor function, NMSs, and sleep disorder characterization of EMO vs. non-EMO. It indicated that EMO is a common symptom in all stages of PD and is associated with higher disease burden and poor QOL in Chinese PwPD, and that sleep disorders may play a contributing role to EMO. This study established that nocturnal sleep problems mediate the detrimental effects on QOL in PD caused by EMO. These findings are important to create more awareness of EMO, screen for EMO, and especially sleep disorders in clinical practice. Further research should confirm causality and the impact on sleep interventions in EMO.

## Data Availability Statement

The original contributions generated for this study are included in the article/Supplementary Material, further inquiries can be directed to the corresponding author/s.

## Ethics Statement

The studies involving human participants were reviewed and approved by the Research Ethics Committee of Xin Hua Hospital affiliated to Shanghai Jiao Tong University School of Medicine. The patients/participants provided their written informed consent to participate in this study.

## Author Contributions

YuZ and ZZ: analysis and interpretation of data and drafting and critical revision of the manuscript. SG, YiZ, LH, YaZ, HW, JZ, FW, CZ, DS, and WW: enrollment of patients and critical revision of this manuscript. DH and ZL: critical revision of this manuscript and supervision of this study. For the detailed information for members of the Shanghai Parkinson's Disease Study Group, see Supplementary Material 1. All authors contributed to the article and approved the submitted version.

## Conflict of Interest

The authors declare that the research was conducted in the absence of any commercial or financial relationships that could be construed as a potential conflict of interest.

## Publisher's Note

All claims expressed in this article are solely those of the authors and do not necessarily represent those of their affiliated organizations, or those of the publisher, the editors and the reviewers. Any product that may be evaluated in this article, or claim that may be made by its manufacturer, is not guaranteed or endorsed by the publisher.

## References

[B1] Ascherio A. Schwarzschild M. A. (2016). The epidemiology of Parkinson's disease: risk factors and prevention. Lancet Neurol. 15, 1257–1272. 10.1016/S1474-4422(16)30230-7 27751556

[B2] Chapuis S. Ouchchane L. Metz O. Gerbaud L. Durif F. (2005). Impact of the motor complications of Parkinson's disease on the quality of life. Move. Disord. 20, 224–230. 10.1002/mds.20279 15384126

[B3] Chaudhuri K. R. Martinez-Martin P. Brown R. G. Sethi K. Stocchi F. Odin P.. (2007). The metric properties of a novel non-motor symptoms scale for Parkinson's disease: results from an international pilot study. Move. Disord. 22, 1901–1911. 10.1002/mds.21596 17674410

[B4] Chinese guidelines for the Treatment of Parkinson′s disease (fourth edition). (2020). Chin. J. Neurol. 53, 973–986. 10.3760/cma.j.cn113694-20200331-00233

[B5] Gómez-Esteban J. C. Tijero B. Somme J. Berganzo K. (2011). Impact of psychiatric symptoms and sleep disorders on the quality of life of patients with Parkinson's disease. J. Neurol. 258, 494–499. 10.1007/s00415-010-5786-y 20957384

[B6] Han C. Mao W. An J. Jiao L. Chan P. (2020). Early morning off in patients with Parkinson's disease: a Chinese nationwide study and a 7-question screening scale. Transl Neurodegener. 9, 29. 10.1186/s40035-020-00208-z 32624000 PMC7336490

[B7] Postuma R. B. Berg D. Stern M. Poewe W. Olanow C. W. Oertel W.. (2015). MDS clinical diagnostic criteria for Parkinson's disease. Mov. Disord. 30, 1591–1601. 10.1002/mds.26424 26474316

[B8] Ray Chaudhuri K. Qamar M. A. Rajah T. Loehrer P. Sauerbier A. Odin P.. (2016). Non-oral dopaminergic therapies for Parkinson's disease: current treatments and the future. NPJ Parkinsons Dis. 2, 16023. 10.1038/npjparkd.2016.23 28725704 PMC5516582

[B9] Rizos A. Martinez-Martin P. Odin P. Antonini A. Kessel B. Kozul T. K.. (2014). Characterizing motor and non-motor aspects of early-morning off periods in Parkinson's disease: an international multicenter study. Parkinsonism Related Disord. 20, 1231–1235. 10.1016/j.parkreldis.2014.09.013 25269446

[B10] Rizos A. Martinez-Martin P. Odin P. Antonini A. Kessel B. Kozul T. K.. (2016). The impact of early morning off in Parkinson's disease on patient quality of life and caregiver burden. J. Neurol. Sci. 364, 1–5. 10.1016/j.jns.2016.02.066 27084204

[B11] Schrempf W. Brandt M. D. Storch A. Reichmann H. (2014). Sleep disorders in Parkinson's disease. J. Parkinsons Dis. 4, 211–221. 10.3233/JPD-130301 24796235

[B12] Stacy M. (2010). The wearing-off phenomenon and the use of questionnaires to facilitate its recognition in Parkinson's disease. J. Neural. Transm. (Vienna). 117, 837–846. 10.1007/s00702-010-0424-5 20563826

[B13] Stacy M. Bowron A. Guttman M. Hauser R. Hughes K. Larsen J. P.. (2005). Identification of motor and nonmotor wearing-off in Parkinson's disease: comparison of a patient questionnaire versus a clinician assessment. Move. Disord. 20, 726–733. 10.1002/mds.20383 15719426

[B14] Stefani A. Högl B. (2020). Sleep in Parkinson's disease. Neuropsychopharmacology. 45, 121–128. 10.1038/s41386-019-0448-y 31234200 PMC6879568

[B15] Stocchi F. Coletti C. Bonassi S. Radicati F. G. Vacca L. (2019). Early-morning OFF and levodopa dose failures in patients with Parkinson's disease attending a routine clinical appointment using Time-to-ON Questionnaire. Euro. J. Neurol. 26, 821–826. 10.1111/ene.13895 30585679

[B16] Stocchi F. Fossati C. Torti M. (2015). Rasagiline for the treatment of Parkinson's disease: an update. Expert Opin. Pharmacother. 16, 2231–2241. 10.1517/14656566.2015.1086748 26364897

[B17] Tysnes O. B. Storstein A. (2017). Epidemiology of Parkinson's disease. J. Neural. Transm. (Vienna). 124, 901–905. 10.1007/s00702-017-1686-y 28150045

